# Re‐constructing nutritional history of Serengeti wildebeest from stable isotopes in tail hair: seasonal starvation patterns in an obligate grazer

**DOI:** 10.1002/rcm.7572

**Published:** 2016-06-09

**Authors:** K. Rysava, R. A. R. McGill, J. Matthiopoulos, J. G. C. Hopcraft

**Affiliations:** ^1^ Institute of Biodiversity, Animal Health and Comparative Medicine, Graham Kerr BuildingUniversity of GlasgowGlasgowG12 8QQUK; ^2^ NERC Life Sciences Mass Spectrometry FacilityScottish Universities Environmental Research CentreEast KilbrideGlasgowG75 0QFUK

## Abstract

**Rationale:**

Nutritional bottlenecks often limit the abundance of animal populations and alter individual behaviours; however, establishing animal condition over extended periods of time using non‐invasive techniques has been a major limitation in population ecology. We test if the sequential measurement of δ^15^N values in a continually growing tissue, such as hair, can be used as a natural bio‐logger akin to tree rings or ice cores to provide insights into nutritional stress.

**Methods:**

Nitrogen stable isotope ratios were measured by continuous‐flow isotope‐ratio mass spectrometry (IRMS) from 20 sequential segments along the tail hairs of 15 migratory wildebeest. Generalized Linear Models were used to test for variation between concurrent segments of hair from the same individual, and to compare the δ^15^N values of starved and non‐starved animals. Correlations between δ^15^N values in the hair and periods of above‐average energy demand during the annual cycle were tested using Generalized Additive Mixed Models.

**Results:**

The time series of nitrogen isotope ratios in the tail hair are comparable between strands from the same individual. The most likely explanation for the pattern of ^15^N enrichment between individuals is determined by life phase, and especially the energetic demands associated with reproduction. The mean δ^15^N value of starved animals was greater than that of non‐starved animals, suggesting that higher δ^15^N values correlate with periods of nutritional stress.

**Conclusions:**

High δ^15^N values in the tail hair of wildebeest are correlated with periods of negative energy balance, suggesting they may be used as a reliable indicator of the animal's nutritional history. This technique might be applicable to other obligate grazers. Most importantly, the sequential isotopic analysis of hair offers a continuous record of the chronic condition of wildebeest (effectively converting point data into time series) and allows researchers to establish the animal's nutritional diary.

Diet is a highly influential aspect of an animal's life, affecting not only nutritional condition, but also morphology, behaviour and consequently the manner in which an individual interacts with its environment.^1^ Populations of many animal species are known to be regulated by food availability (bottom‐up regulation), leading to the evolution of various coping mechanisms, such as decreased activity levels to conserve energy or increased activity levels to search for new resources.^2^, ^3^, ^4^ Yet, thus far, it has not been possible to study the continuous dynamics of nutritional stress, as a longitudinal dataset, over extended periods. Hence, the exact life‐history trade‐offs that have propelled the diversification of various life‐strategies remain unclear. For example, differentiating the proximate versus ultimate causes that limit animal populations would enable ecologists to quantify how animals balance the demands of energetically challenging life‐phases with the risk of starvation.

Currently, most ecological studies of diet and nutritional condition are limited to direct field observations^5^, ^6^ that are too complicated to perform with a consistent level of quality, especially in the case of highly mobile or elusive animals. Resource selection studies based on observational data, for example, provide an index of preference for habitats or food types; however, they do not account for an animal's condition over long periods of its life. In order to address the lack of knowledge regarding the body condition over time, alternative approaches need to be considered.

Stable isotope analysis allows ecologists to infer the quality and composition of animals' diet and describe their spatial distributions.^7^ Stable isotopes from various body tissues and faeces 8 have been used in most chordate classes to investigate trophic interactions, dietary preferences and seasonal dietary shifts.^5^, ^7^, ^9^ However, repeated time‐sequenced analyses of focal animals are not common and measurement techniques are often invasive using tissues such as teeth, bone or muscle.^10^, ^11^ Hair may provide a good alternative source of information about the physiological condition of live animals because it can be collected non‐invasively, it grows continuously, and it may retain relevant metabolites from which the chronic condition of the animal can be inferred over long periods of time.^12^ Previous studies have illustrated that tail hair retains isotopic information and can be used to understand an animal's history, such as the large‐scale movement and dietary preferences of Asiatic wild ass (*Equus hemionus*) in the Mongolian Gobi^13^ or the dietary overlap of endangered Grevy's zebra (*Equus grevyi*) with livestock in Laikipia, Kenya.^14^ However, despite its potential, nitrogen stable isotope analysis of hair tissue has not been fully exploited as a metric of individual body condition primarily because it has not been adequately quantified.

The migration of Western white‐bearded wildebeest (*Connochaetes taurinus*) in the Serengeti provides a good case‐study to quantify the use of stable isotopes in a highly synchronous, bottom‐up regulated population of terrestrial migrants. The entire population of approximately 1.3 million wildebeest seasonally migrates in a circular pattern across a rainfall and soil fertility gradient in search of fresh grazing.^15^ Starvation is the primary cause of mortality in this population which is limited by food,^16^ especially when the rains fail after a prolonged period of drought, and intraspecific competition for resources is intense among wildebeest individuals.^17^ Although nitrogen isotope ratios (given as δ^15^N values) typically indicate switches between different types of forage and trophic interactions,^18^ for obligate grazers such as wildebeest that do not switch between different types of food^19^ changes in nitrogen isotope ratios may not be a consequence of diet shifts. Hobson *et al.* 20 hypothesized an alternative mechanism of ^15^N enrichment induced by nutritional stress: in this case, variation in the enrichment of ^15^N over the length of the wildebeest tail hair could be related to switches in the overall availability of food such that free nitrogen in the body is acquired from the animal's forage during periods of plenty whereas it is catabolized from the body's muscle tissue and fat stores during periods of paucity. Therefore, ^15^N enrichment could be a potential indicator of starvation^21^ in this obligate grazing species. If the δ^15^N value is indeed a reliable indicator of the seasonal energy demands and starvation, δ^15^N values should vary between individuals in different life‐history stages and within the same individuals over time.

This study investigates if the enrichment of ^15^N in the tail hair of Serengeti wildebeest provides information about an animal's nutritional condition, which could enable ecologists to re‐construct the starvation history of an animal over time. Specifically, we ask two questions:

1. Is there temporal variation in the δ^15^N value along the length of the tail hair and is it consistent between contemporaneous tail hairs from the same individual (i.e. the approach is repeatable if there is little intra‐individual variation)? A similar sequence of δ^15^N values from different strands of hair from the same animal would suggest that the growth of tail hair is uniform and that isotopic enrichment is consistent across all hairs.
2. Are values of δ^15^N in the tail hair an accurate proxy for starvation? If a high δ^15^N value is correlated with episodes of chronic starvation, we expect (a) the hair segments that are grown during periods of high energy output (such as reproduction or lactation when animals may begin to catabolise their muscle tissue) to have higher δ^15^N values than when animals are not nutritionally stressed. Furthermore, (b) we expect the most recent δ^15^N values to be higher in animals that have died of starvation than in animals that have died of other causes, and (c) we expect the annual cycle of δ^15^N values to differ between males and females because of the extreme energy demands of reproduction on females. However, if the δ^15^N value simply reflects the isotopic value of the forage that is available, we expect both males and females to have similar annual patterns of δ^15^N variation because they feed in the same areas as they migrate.

## Experimental

### Sample collection

Wildebeest tail hair was collected from the Serengeti Mara ecosystem, East Africa (1°15' to 3°30'S, 34° to 36°E) between 2012 and 2014. We sampled the tail hair from 15 wildebeest individuals in total; 14 females and 1 male. Tail hair was collected either from carcasses of animals that had died of natural causes (10 females and 1 male) or from live animals that were temporarily immobilized while deploying GPS radio collars (4 females). The current maternal status was recorded for the live females (with or without calf), and the cause of death was determined for all carcasses (starved versus non‐starved) by classifying the state of the bone marrow in the femur. Mammals mobilize the fat stores in the bone marrow during the final stages of starvation and hence its colour and consistency can be used as a reliable indicator of the body condition preceding death (bone marrow liquefies and transitions from creamy‐white to opaque as starvation proceeds^22^). The combination of samples allowed us to test our hypotheses regarding animal condition and energy expenditure by dividing the individuals into the following five groups: (i) females that died of starvation (n individuals = 4; n samples = 83), (ii) females that died of other causes (n individuals = 4; n samples = 84), (iii) females with a dependent calf (n individuals = 3; n samples = 63), (iv) females without a calf (n individuals = 3; n samples = 63; note that 2 of the 3 samples were sub‐adult females and hence reproductively inactive for their entire lives), and (v) a single male (n individuals = 1; n samples = 19) as an outgroup for comparative purposes. Fresh hair samples were washed in water in the field before being stored in paper envelopes and transferred to the lab.

### Sample preparation and stable isotope analysis

Sample preparation and stable isotope analyses were conducted at the Natural Environment Research Council's Life Sciences Mass Spectrometry Facility (East Kilbride, UK). Prior to any processing, hair samples were cleaned from any impurities, urea and lipids using a methanol bath and wipe. We adopted the protocol of Mekota *et al*.,^21^ but instead of a 2:3 mixture of methanol and chloroform we used a pure methanol solution since our samples did not contain any flesh or blood residue.

To test the hypothesis of intra‐individual variation in nitrogen isotopic values, we created three bundles of tail hair from the same individual (bundles A, B and C) consisting of three hair strands each, from two non‐starved females. All bundles were aligned by the roots to a maximum length of 32 cm, representing approximately 18 months of an animal's life (growth rate approximation derived from repeated hair measurements of recaptured wildebeest individuals and measurements of tail hair length from multiple carcasses of known ages from birth to adulthood [0 to 3 years old], unpublished data). We clipped each bundle into 40 segments, each 8 mm long, which corresponded to approximately 2 weeks of the animal's life. Every second segment was minced with surgical scissors and 0.70 mg (±0.05 mg) of the sample was packaged into tin capsules for isotopic analysis. Each 8‐mm hair segment was treated as a separate observation, such that each of the 3 bundles from the 2 individuals had 19 consecutive samples, giving a total of 57 samples per wildebeest.

To test the hypothesis regarding the temporal sequence of starvation, approximately 200 strands of cleaned tail hair from each individual were aligned by the roots and secured into a bundle by embedding the roots into a plug of epoxy glue. The bundles were clipped into 8‐mm long segments. Every second 8‐mm segment of hair was homogenized by placing it into a 1.5‐mL round‐bottomed Eppendorf vial with two Retsch cone balls (3 mm), snap frozen in liquid nitrogen for 2 min, and homogenized using a Retsch MM 301 mixing mill (Retsch UK Ltd, Hope Valley, UK) at 25 oscillations/s for 9 min. A sample of ground hair weighing 0.70 mg (±0.05 mg) was packaged into a tin capsule for isotope analysis.

All samples were analysed using an ECS 4010 elemental analyser (Costech International S.p.A., Milan, Italy) coupled to a Delta V Plus isotope ratio mass spectrometer (Thermo Fisher Scientific, Bremen, Germany). Laboratory standards, Fluka gelatine, Sigma alanine and Sigma glycine, (Sigma‐Aldrich Company Ltd, Gillingham, UK) were repeated with every 10 samples and were used to correct for linearity and instrument drift over a 16‐h analytical run. The isotope ratios for lab standards are determined relative to a range of International standards (Table 1) from IAEA (Vienna, Austria) and USGS (Reston, VA, USA). The analytical precision for nitrogen isotopes was better than 0.3‰.

**Table 1:** δ^15^N values of the tryptophan (lab standard) and USGS40 (international standard): mean and standard deviation for each day

Tryptophan		USGS40	
mean	sd	mean	sd
–2.71	0.07	–4.83	0.13
–2.07	0.03	–4.09	0.07
–2.23	0.05	–4.21	0.24
–2.07	0.03	–4.23	0.25
–2.1	0.08	–4.23	0.27
–2.02	0.08	–4.06	0.06
–2.11	0.06	–4.16	0.14
–2.07	0.03	–4.23	0.25
–2.07	0.03	–4.09	0.07
–1.98	0.17	–3.93	0.17
–2.58	0.13	–4.58	0.04
–2.64	0.1	–4.71	0.14
–2.23	0.05	–4.21	0.24
–2.1	0.08	–4.23	0.27

n = 4 for both the standards for each day.

Measurements are independent of any correction calculations.

The isotopic ratios are expressed in the δ (delta) notation in parts per million (‰):

$$\mathrm{\delta X}=\left[\left(R_{\text{sample}}/R_{\text{standard}}\right)–1\right]$$

where X = ^15^N and R = the ratio of ^15^N/^14^N isotopes in a given sample compared with AIR.

### Statistics and data analysis

Generalized Linear Models (GLM) in R version 3.1.2^23^ were used to determine if there was a significant difference in the time‐series of δ^15^N values between the three bundles of hair from the same individual (i.e. the test of intra‐individual variation). Specifically, the GLM tested if the variation in δ^15^N values between concurrent segments of hair (section 1, 3, 5 …19) was a function of the bundle identification – A, B or C (2 individuals, n = 59 and 60 each; sample size per group = 19 and 20). The premise that the δ^15^N value is consistent between collateral tail hairs would be supported if the bundle identification failed to account for the difference in the δ^15^N values for each concurrent hair segment between bundles.

The difference between the δ^15^N values from individuals that died of starvation as opposed to those that had died of other causes was evaluated by selecting the hair segments from the last month of an animal's life (sections 1 and 3 from the root end), as it is unlikely that a wildebeest can survive on stored body fat for more than a month. If ^15^N enrichment is correlated with starvation episodes, the δ^15^N values should be greater in starved animals than in non‐starved animals. The variation in δ^15^N values in relation to the cause of death was tested using Generalized Linear Models, where the δ^15^N value was a function of whether the animal starved to death or not (n = 32; sample size per group = 16). We also performed a power analysis on the dataset to calculate the minimum difference in δ^15^N values between the groups given the available sample size at a 5% significance level with 95% confidence and 80% power.

The energy demands of wildebeest are likely to behave non‐linearly over time; therefore, Generalized Additive Mixed Models (GAMMs) were used to test if periods of greatest energy demand during the annual cycle of wildebeest were correlated with ^15^N enrichment in the tail hair. Previous research has shown an inverse relationship between body fat (measured in kidneys and bone marrow) and lactation (weight of the mammary glands) in wildebeest, suggesting that nursing is the most energetically demanding time for female wildebeest.^24^ Furthermore, wildebeest in the Serengeti are highly synchronous breeders; therefore, the entire population of reproductively active females consistently reaches peak lactation in March to May every year.^25^

GAMMs were implemented in R version 3.1.2^23^ using the package mgcv.^26^ The GAMM framework allows for multivariate modelling where the response to any particular covariate (or combination of covariates) can be smoothed to account for non‐monotonic patterns in the data. A GAMM fitted to the annual cycle of δ^15^N values in the tail hair of lactating female wildebeest (n = 63) was used to quantify the explanatory power of the following predictor variables: Julian day (JD), implemented using a cyclic spline from day 1 to day 366 for each year of the study; age, estimated from tooth wear (with no smoothing function applied); and individual as a random effect. The existence of residual temporal autocorrelation was also tested by embedding into the GAMM an AR1 model (an autoregressive model of order one) for the data from each individual, separated by Days (form = ~DAYS|INDIVIDUAL). If the δ^15^N value is correlated with periods of high energy demand, the partial residuals of Julian day on the δ^15^N value should be greater than zero during the months of peak lactation (March to May) and less than zero when the animals stop lactating (September) for all lactating females.

The mean monthly ^15^N enrichment between lactating females and the one male was compared using standard boxplots. If the δ^15^N value is correlated with the varying energy demands of the animal, the δ^15^N value in males should be highest during the rut when male competition for females is at its peak (July). Previous research indicates that males often forego grazing during the rut,^27^ resulting in decreased kidney and bone marrow fat.^28^ However, if ^15^N enrichment is a function of the local quality of the forage rather than the energy expenditure of the individual, (a) the annual pattern of δ^15^N values of the male and the females should be the same because they have similar migratory patterns and (b) their δ^15^N values should be identical in July because both sexes occupy the same location during the rut.

## Results

### Intra‐individual variation

**Figure 1:**
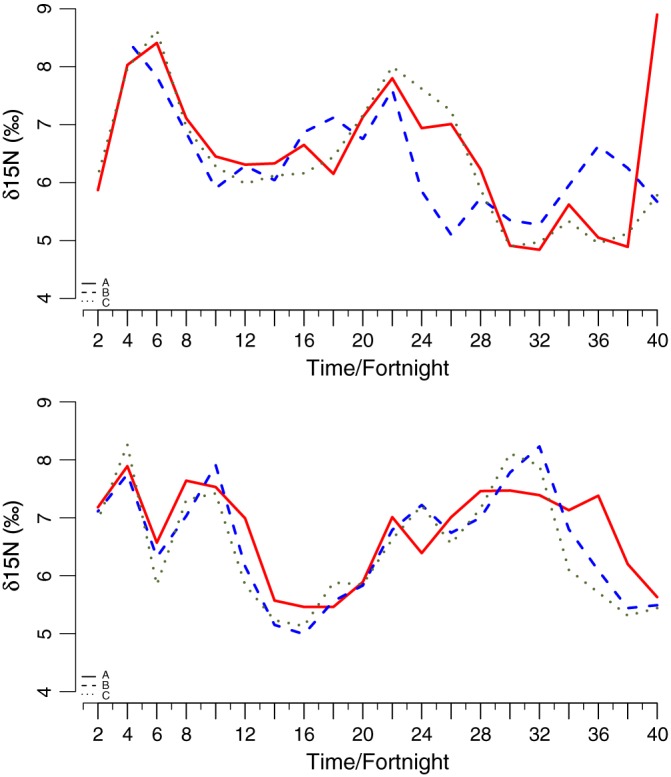
Comparison of δ^15^N values extracted from the concurrent segments of tail hair from the same individual (bundles A, B and C) plotted against time (expressed in fortnight intervals from an animal's day of death). Each line represents a hair bundle consisting of three hairs strands each: (a) individual 30 and (b) individual 59.

The GLM analyses comparing the variation of δ^15^N values within each segment of hair across three replicate bundles of hair from the same individual failed to include bundle identification in the model, suggesting low intra‐individual variation. Figure 1 shows several small discrepancies between bundles for both individuals, but every bundle tends to follow the same annual pattern. Statistically there was no significant difference between bundles: p‐values comparing bundle A versus B and A versus C were 0.694 and 0.657, respectively, for the first individual (Fig. 1(a); residual deviance = 64.219 on 56 degrees of freedom) and 0.512 and 0.359 for the second individual (Fig. 1(b); residual deviance = 48.674 on 57 degrees of freedom).

### Starved versus non‐starved animals

**Figure 2:**
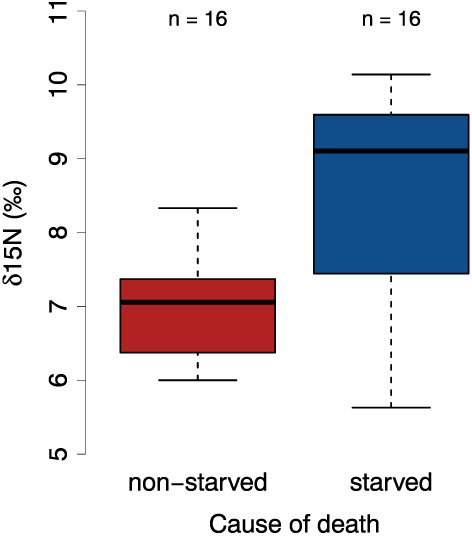
Boxplot of δ^15^N values relevant to the last month of animals' lives preceding death. Animals that died of starvation (in blue, n = 8) exhibit proportionally higher δ^15^N values than individuals that died as result of a non‐starvation event (in red, n = 8).

The variation in δ^15^N values from the tail hair of dead individuals is partially explained by the cause of death (starved versus non‐starved; residual deviance = 23.389, 14 degrees of freedom, p <0.05). Figure 2 demonstrates the differences between the δ^15^N values of the starved and non‐starved groups, showing substantially higher δ^15^N values for the starved animals. The results from the power analysis suggest that given our sample size the minimum difference in the means between the two groups needs to be at least 0.97 δ^15^N ‰ for it to be significant (standard deviation = 0.5, p = 0.05). The actual difference in mean δ^15^N values between the starved (8.5 δ^15^N ‰) and non‐starved (6.9 δ^15^N ‰) groups is 1.6 δ^15^N ‰, which is above the recommended minimum value suggesting there is no reason to be concerned about the sample size (Fig. 2).

### Starvation and energy expenditure

The GAMM model investigating variation in the annual cycle of δ^15^N values in the tail hair revealed that 'Julian day' was the only significant predictor (r^2^ = 0.301, n = 63, p <0.001). The age of the animal was borderline in explaining the variance of δ^15^N values in lactating females (p = 0.065). Furthermore, the cyclic smooth term on 'Julian day' was significant (p <0.001). An independent model was preferred over a model with temporal autocorrelation (AIC values = 182.98 for the independent model and 184.98 for the autocorrelation model) and the redundancy of autoregressive structure was further supported (φ = 0, suggesting that the δ^15^N value at time t is independent from t–1).

**Figure 3:**
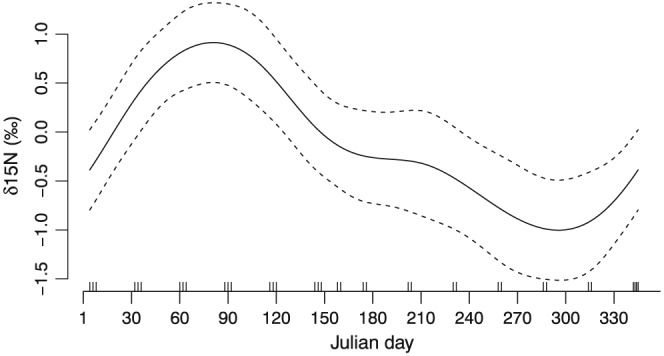
The estimated residuals from the GAMM of Julian day on δ^15^N values, suggesting that the δ^15^N value follows a predictable annual cycle. Each time sample point is indicated by a tick mark at the bottom of the graph.

The partial residuals of Julian day on the δ^15^N values of lactating females illustrate the strong annual cycle across the length of the hair (Fig. 3). The δ^15^N value rapidly increases above zero by the 50^th^ Julian day (February), reaching its peak by the 75^th^ Julian day (mid‐March). The trend then becomes negative, and the trajectory drops below zero by the 150^th^ Julian day (the end of May) and continues to decline until it reaches the lowest point by the 300^th^ Julian day (October). After this point the δ^15^N values of lactating wildebeest females start to increase again through November and December.

**Figure 4:**
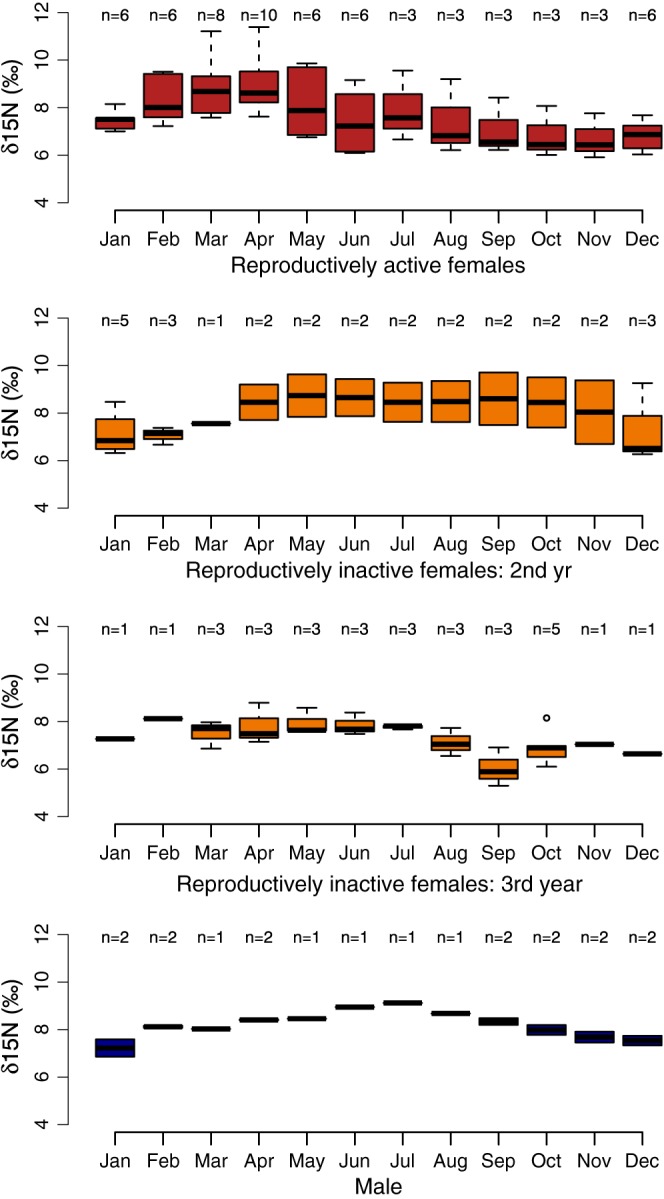
The average monthly δ^15^N values for (a) reproductively active females (n = 3), (b) reproductively inactive female yearlings (n = 3), (c) reproductively inactive females in their third year of life (n = 2), and (d) a male (n = 1) shows different annual cycles.

The monthly mean δ^15^N values for all reproductively active females show peaks in February to May when the variance also tends to be greatest (Fig. 4(a)). The δ^15^N value was lowest from September to November. The monthly mean δ^15^N value for the male was different from that of the reproductively active females; the male's annual cycle of δ^15^N values reached its maximum in July (Fig. 4(c)) whereas the female δ^15^N value was declining during this time period.

## Discussion

The results suggest that the sequential analysis of stable isotopes in hair may be a useful non‐invasive method for determining the physiological condition and nutritional history of individuals over long periods of time and could help expand our current understanding of the population dynamics of wild animals. For instance, these techniques could provide deeper insights into observed patterns of habitat use and seasonal modes of population regulation by identifying critical bottlenecks in the annual chronosequence of animal nutrition.

The main findings from this study are that (a) the longitudinal variation in δ^15^N values is consistent among individual hair strands from the same animal suggesting that hair growth is synchronous and that the approach is repeatable (Fig. 1); (b) enrichment of ^15^N is correlated with periods of starvation (Fig. 2); and (c) the annual cyclic variation of ^15^N enrichment is similar among individuals with the same life‐histories (Figs. 3, 4). Combined, these results suggest that ^15^N enrichment in the tail hair of obligate grazers is closely correlated with periods of negative energy balance when the animal is forced to mobilize internal reserves (fat and muscle tissues) and that it could be used as a reliable bio‐indicator of the animal's nutritional history. We also tested for temporal autocorrelation in δ^15^N values between consecutive segments of the wildebeest tail hair. Although there was marginal support for temporal autocorrelation in our analysis, if we sampled more frequently (i.e. used every 14‐day section rather than every second 14‐day section [sections 1,2,3…40 rather than 1,3,5, … 39] or reduced the length of each section so that it was equivalent to a week [4 mm rather than 8 mm]) it is likely that there would be significant temporal autocorrelation.

The hypothesis that ^15^N enrichment in the tail hair of wildebeest is correlated with periods of negative energy balance in the body is supported by multiple lines of evidence. First, the comparison of δ^15^N values between animals that died of starvation and those that died of other causes (as differentiated by the colour and consistency of the bone marrow) suggests that the greatest ^15^N enrichment in tail hair is seen in animals that were starving in the month preceding death (Fig. 2). Second, highest ^15^N enrichment in the annual cycle of reproductively active females coincides with periods of peak lactation in March when nursing females experience the most intense energy expenditure, while the lowest δ^15^N values occur from September to October once calves are weaned.^25^ The large variance in monthly mean ^15^N enrichment around peak lactation in March (Fig. 4(a)) is probably caused by differences in calf survival. Previous research suggests that up to 40% of calves die within the first 3 months of life depending on the grazing conditions;^29^ therefore, females who lose their calf may actually gain weight during this time as opposed to losing weight through milk production, thus resulting in increased variation in the δ^15^N response. Information on the breeding status of reproductively active females was certain only at the time of sampling (by direct observation of the calf) and could not be confirmed for sections of the hair that had grown in previous years. It is possible that some of the sampled females had lost their calves or even failed to breed in the preceding year giving a wide range of δ^15^N values in February, March and April. These conclusions agree with our original hypothesis that the tail hair tends to become ^15^N‐enriched during times when herbivores supplement their diet by metabolising their internal fat stores and muscle tissue.

Wildebeest migrate around the Serengeti‐Mara ecosystem in a predictable pattern. Therefore, it is conceivable that the ^15^N enrichment observed in the hair is a product of the animals sequentially moving between different grazing patches each with its own distinctive value (i.e. an exogenous origin of δ^15^N variation rather than endogenously related to starvation). The evidence suggests that this is unlikely: both males and females travel along the same migratory route, but the pattern of annual isotopic variability in the tail hair is not similar between the sexes (Figs. 4(a) and 4(d)) nor between individuals in different phases of their life‐histories (Figs. 4(a), 4(b) and 4(c)). Furthermore, during the rutting season (early to mid‐June) both males and females aggregate at exactly the same locations to mate; however, the δ^15^N values during June differ both between the sexes and between reproductive and non‐reproductive females, suggesting that the signature is not spatially dependent. Interestingly, behavioural observations^27^ show that males rarely eat during the rut and focus almost entirely on protecting their harem from other males, resulting in a loss of body condition.^[25]^ This is corroborated by our observations of enrichment in ^15^N in the male during the rut (June and July in Fig. 4(d)), which further supports the premise that a high δ^15^N value is indicative of negative energy balance rather than being spatially dependent on the nitrogen isotope signature of the forage. Sampling multiple males would be critical to further validate these results. Furthermore, a large‐scale sampling regime of the vegetation across this entire ecosystem would establish if there are distinct spatial isoclines of δ^15^N in the grass and further elucidate the role of forage in the δ^15^N signature of the hair. Alternatively, a comparison between hair samples from migratory and non‐migratory wildebeest populations would allow us to differentiate whether patterns of δ^15^N variability are physiologically or geographically determined
Note there are four small sub‐populations of resident wildebeest in the Serengeti‐Mara ecosystem that do not migrate, but whose home ranges are sympatric with the much larger migrant population.^23^
. In species described as drought‐tolerant, the seasonal ^15^N enrichment in body tissues is hypothesized to be a result of water rather than food limitations, due to isotopic processes occurring during amino acid metabolism associated with water conservation.^30^ Ambrose and DeNiro^31^ suggested that African herbivores physiologically adapted to extended periods with no access to water have higher nitrogen isotope ratios than water‐dependent species. Wildebeest, however, are defined as obligate drinkers^31^ and require free water at least every second or third day; therefore, an increase in δ^15^N values caused by adaptations to water stress is unlikely. This is directly supported by our findings, which show an increase in δ^15^N values in times when wildebeest were not water‐challenged (i.e. the wet season).

The results complement and expand previous findings using inert biological material such as hair, feathers, claws and scales to ascertain information about animal histories, and diet in particular. For example, Cerling *et al.* 32 determined the stable isotope ratios of nitrogen and carbon from hair samples of African elephant (*Loxodonta africana*) in northern Kenya and found that elephants switch between C3 browse and C4 grasses depending on the rains. Similar studies on species such as koala (*Phascolarctos cinereus*),^33^ north‐eastern Pacific white shark (*Carcharodon carcharias*)^34^ and king penguin (*Aptenodytes patagonicus*)^7^ also correlate changes in isotope ratios with diet and the spatial distribution of animals. However, the novelty of using tail hair is that we are able to turn this information into a time‐line that can differentiate between distinct phases of the animal's life over the preceding 2 years. The use of hair as a natural bio‐logger of seasonal energy expenditure and forage quality enables ecologists to gather large amounts of life‐history information from many animals in the population thus expanding critical datasets. Evidently, the strength of the results demonstrated here may be partially limited by the modest sample size of individuals in the study. Increasing the size of our dataset for each category would be the most natural extension to the current work. Furthermore, the expansion of the GAMM framework to include individuals in other life‐history phases in addition to reproductively active females would provide additional insights.

## Future Directions

Inherently, there are ways in which this technique could be further elaborated and improved. First, simultaneous hormone assays (such as progesterone) on each section of hair would minimize the uncertainty about the animal's breeding status (i.e. pregnant or not) and could potentially allow researchers to follow the reproductive history of females with greater accuracy. Simultaneous progesterone assays would also shed light on the relationship between reproductive status and starvation, such as whether starvation acts to limit reproduction or whether reproductive status leads to an increase risk of starvation.^35^ Previous research suggests that progesterone can be detected during early pregnancy in hair samples from cattle.^36^ However, it still remains unclear if steroid hormones can be detected in wildebeest hair, and indeed whether this could be used to re‐create a clear chronological profile of the animal's reproductive history.

The second improvement would be to determine stable isotope ratios for several other elements from the same sequence of samples, thereby broadening the forensic information available regarding the animal's physiology and ecology. For example, the isotopic analysis of carbon could allow inference about dietary preferences (i.e. the ^14^C signature of C3 versus C4 plants), or the timing of dietary overlap in sympatric species (such as wildebeest, plains zebras (*Equus quagga*) and African buffalo (*Syncerus caffer*)).^5^ Furthermore, the unique signature of oxygen and hydrogen isotopes from different water sources could provide a natural bio‐marker of geographic location^37^, ^38^ and systematic variation in δ^2^H and δ^18^O values over the length of the hair might provide valuable insight into the pattern and timing of wildebeest migration. In addition, strontium gradients in the bedrock (^87^Sr/^86^Sr) might provide further information about the seasonal movement of local fauna across the landscape.^39^, ^40^ The sequential analysis of tail hair for strontium, hydrogen and oxygen isotope ratios requires further investigation.

A further development of interest would be to assess endogenous versus exogenous sources of δ^15^N values by analysing specific amino acids from the keratin of the hair.^41^ The carbon skeleton of certain amino acids such as alanine is dispensable (i.e. alanine undergoes *de novo* synthesis from pyruvic acid; its carbon skeleton largely reflects the dietary carbohydrate), while others such as lysine must be ingested as part of the diet of the animal (i.e. indispensable amino acids).^42^ Compound‐specific δ^15^N analyses of non‐essential amino acids in tail hair could differentiate between periods in the animal's life when a smaller proportion of core resources (such as the nitrogen building‐blocks used in making the amino acids) are accessed from their immediate food supply as opposed to being mobilised via the breakdown of protein. By sequentially analysing certain indispensable amino acids, the δ^15^N values of the food supply could be differentiated from the values generated through internal processes. The fractionation and sequential isotopic analysis of essential and non‐essential amino acids over the length of the tail hair would unequivocally resolve if the patterns of ^15^N enrichment that we observed are indeed a true reflection of the balance between amino acid supply and the demands on the animal over time (Tom Preston, University of Glasgow; personal communication).

## Conclusions

The results suggest that nitrogen isotope ratios in longitudinally analysed tail hair are consistent between strands and that the δ^15^N values can be used to re‐construct the nutritional condition in obligate grazers over long periods of time. The data from isotopic analysis of sequentially sampled tail hair could offer a continuous record that allows inference about the chronic physiological condition of an animal over time and greatly expands our insights into animal physiology and ecology. The novelty of this approach is that it could allow ecologists to forensically establish the sequence of events prior to the observation (such as the condition of the animal in the months preceding death), thereby expanding point observations into time‐series data. This technique could be used to investigate a wide range of ecological questions such as the relative physiological costs and benefits of migratory and non‐migratory behavioural strategies, or the energy expenditure and nutritional bottle‐necks of competing sympatric species. Moreover, it could also be employed in wildlife forensics to inform about the role of nutritional stress during epidemics or the duration of malnutrition in mistreated animals. The full realisation of any tool, however, relies on its ability to be utilised in future research and management. Expanding this technique to simultaneously include other isotopes and metabolites could assist researchers to meet the challenges in describing and understanding complex systems.
